# The pathogenesis of optic neuritis caused by *Angiostrongylus cantonensis* in BALB/c mice

**DOI:** 10.1186/1756-3305-7-339

**Published:** 2014-07-22

**Authors:** Ying Feng, Xin Zeng, Wei-Hua Li, Wen-Cong Wang, Wei Chen, Li-si Ou-Yang, Xi Sun, Feng Feng, Zhong-Dao Wu

**Affiliations:** 1 Zhongshan School of Medicine, Sun Yat-sen University, Guangzhou 510080, China; 2 Key Laboratory of Tropical Disease Control (SYSU), Ministry of Education, Guangzhou 510080, China; 3 Zhongshan Ophthalmic Center, Sun Yat-sen University, Guangzhou 510080, China; 4 The twelfth people's Hospital of Guangzhou City, Guangzhou 510080, China

**Keywords:** Optic neuritis, Infection, *Angiostrongylus cantonensis*, BALB/c mice

## Abstract

**Background:**

One of the most common causes of meningitis in South East Asia is angiostrongyliasis or infection by the parasitic nematode *Angiostrongyliasis cantonensis.* Although this nematode usually resides in the pulmonary arteries of rats, its incidental occurence in other hosts such as humans can cause optic neuritis and lead to serious vision sequelae. Nevertheless, there are currently no systematic studies conducted in this area.

**Methods:**

In order to study the pathogenesis of optic neuritis, mice were tried as a new animal model to study and challenge with *A. cantonensis* on 7d, 14d and 21d, respectively. Electroretinogram (ERG), visual evoked potential (VEP), ophthalmoscopy and histology were examined on day 7d, 14d and 21d and tribendimidine (TBD) was later used to treat optic neuritis on day 14d for a week to evaluate its therapeutic effects.

**Results:**

Infection of *A. cantonensis* caused obvious inflammatory cell infiltration in the retina and optic nerve adventitia in day 14d and 21d followed by optic nerve fiber demyelination and retinal ganglion swelling at day 21d in the challenged mice. Prolonged VEP latency and decreased ERG amplitude were also observed on day 21. After treatment of TBD in the infected mice, retinal and optic nerve inflammation were alleviated, but VEP latency and ERG amplitude did not improve on day 21d and 28d.

**Conclusions:**

The current study provides evidence that *A. cantonensis* can cause optic neuritis along with optic nerve demyelination and retinal ganglion cell damage in a mouse model. TBD alone treatment can improve the symptoms of optic neuritis, but does not aid in vision recovery, suggesting that both neuroprotective agents and Dexamethasone should be administered, along with treatment for the infection, to protect the optic nerve and ganglion cells. Furthermore, as the symptoms of optic neuritis caused by *A. cantonensis* in mice are similar to the optic neuritis in multiple sclerosis (MS) human patients, we suggest that the BALB/c mouse model provided in this study may be useful to explore therapies of optic neuritis in MS patients.

## Background

*Angiostrongylus (A). cantonensis* is a parasitic nematode that causes Angiostrongyliasis (also called Eosinophilia Meningoencephalitis or Eosinophilia Myeloencephalitis). Fresh water snails harbor the third stage of the *A. cantonensis* larva (Larva), which is the infective stage. Consumption of raw or uncooked snails or fish is the most common manner, resultant in infection by *A. cantonensis*. In humans, 3^rd^ stage larva mainly invade the central nervous system, causing meningitis, spinal meningitis, encephalitis and myelitis. The clinical manifestation of infection is eosinophilic meningitis [1-5], which can affect the eyes to cause optic neuritis. There are ongoing reports of optic neuritis cases caused by *A. cantonensis*[6]. The first case of this disease was reported in Thailand in 1966 [7]. Since then, multiple cases have been reported. In 2006, a patient presented with sudden vision loss along with visual field, color vision, and visual evoked potential (VEP) deficiencies and was found to have midrange optic neuritis [8]. In 2008, Shindler *et al*. conducted a clinical study of 3 optic neuritis cases caused by *A. cantonensis.* Larvae were found in the anterior chamber angle, vitreous body and retina and *A. cantonensis* antibody (29 KD) was detected in serum. Patients underwent visual acuity, random amplified polymorphic DNA (RAPD), VEP, and electroretinogram (ERG) testing as well as fundus examinations [9]. In 2006, a small outbreak of *A. cantonensis* in Beijing, China, caused the attention of the Chinese government on the ponderance of *A. cantonensis*. In one survey about this outbreak, 25 severe cases were identified, 44% of which had different levels of visual disorders, such as photophobia, blurred vision, diplopia, visual field defects and/or muscae volitantes (‘floaters’). Even after treatment, a fairly substantial proportion of patients did not recover, seriously affecting quality of life [10,11]. Up to now, there have been few reports on the mechanism(s) by which *A cantonensis* causes optic neuritis.

The purpose of the present study was to establish a mouse model of optic neuritis caused by *A cantonensis* to elucidate a pathogenetic mechanism of the disease and to develop therapeutic strategies for clinic treatment. In this study, mice that had been infected with *A. cantonensis* were used as a surrogate model to observe optic neuritis. Obvious VEP/ERG changes and pathological alterations of retina and optic nerve, such as retinal ganglion swelling and optic nerve demyelination, were observed in the infected mice. The effect of treatment with a new anthelmintic agent, Tribendimidine (TBD) [12] was also examined. Our results indicate that TBD eases inflammation in the retina and optic nerve, but vision is not improved. Our studies demonstrate that infection of *A. cantonensis* is able to cause optic neuritis. It is necessary to develop new drugs, which can protect and nourish optic nerve and ganglion cells during the treatment of infection [13].

## Methods

### Infection of mice with *A. cantonensis* larvae

BALB/c mice (20 – 40 g body weight) were purchased from the animal center laboratory at Sun Yat-sen University (Guangzhou, China). The Institutional Animal Care and Use Committee approved all animal procedures. Larva III (L3) of *A. cantonensis*, were collected from giant African snails (*Achatina fulica*) via homogenization and digestion of minced snail tissue that was placed in a pepsin–HCl solution (pH 2.0, 500 IU pepsin/gram tissue) and incubated at 37°C for 2 h. The L3 in the sediment were washed with phosphate-buffered saline (PBS) and counted under an anatomical microscope. Four groups (n = 6 mice/group) were studied. In the 3 mice of each group, a total of 30 L3 were perfused into each BALB/c mouse; the other 3 mice in each group were not infected and served as controls. At the same time, infected animals were randomly assigned to either the treatment or non-treatment group (n = 5 animals or 10 eyes in each group). TBD was administered via gastric infusion to mice in the treatment groups once per day for 7 days. The dose of TBD was 100 mg/kg/d [14]. TBD was a gift from Professor Guosheng He, Shanghai Veterinary Research Institute, Chinese Academy of Agricultural Sciences.

### Histopathological observation

Mice were sacrificed by chloral hydrate asphyxiation and cervical dislocation at 7, 14, or 21 days post-infection. Paraffin embedded sections were then prepared from isolated optic nerves and whole eyes. Optic neuritis was detected by the presence of inflammatory cell infiltration on hematoxylin and eosin (H&E) staining and observed by microscopy (Olympus, Japan). Retinal thickness was measured using Image Pro Plus 6.0 (Media Cybernetics, USA). Demyelination detection was done by using a 300 KV transmission electronic microscope (FEI, USA) and axons were counted with Image Pro Plus 6.0.

### Electrophysiological recordings

Mice in all four groups were anesthetized by intraperitoneal injection of chloral hydrate (0.42 mg/kg body weight) and secured to a moveable platform for ERG and VEP testing. The Flash ERG was recorded with chlorinated silver ball electrodes as described previously [15]. For VEP recordings, stainless steel screws were implanted 3 mm lateral to the lambda and 5 mm behind the bregma. Reference electrodes were placed 2 mm lateral to the lambda and 2 mm in front of the bregma. Transient VEPs were evoked by single-flash stimulation (1.3 Hz, 12 ms). VEP and ERG signals were recorded by a commercial system (Roland Consult GmbH, Brandenburg, Germany). Recordings were performed at day 0, 7, 14, and 21 after infection.

### Retrograde labeling of retinal ganglion cells (RGCs)

Two weeks before euthenasia, mice were anaesthetized with chloral hydrate, the skin was incised mediosagittally, holes were drilled into the skull above each superior colliculus (6.8 mm dorsal and 2 mm lateral from the bregma) and 0.25 μl of Dextran biotin (A and B: 1:250 in normal saline) (Molecular Probes, USA) was injected stereotactically into both superior colliculi.

### Quantification of retinal ganglion cell (RGC) density

At the conclusion of the second recording session, the mice were euthanized by an overdose of chloral hydrate and were perfused via the aorta with 4% paraformaldehyde in PBS. The brain, optic nerves and both eyes were collected. The retinas were then dissected and soaked in horseradish peroxidase (HRP) at 37°C for 30 min. Retinas were then stained for 5 min with 3,3'-diaminobenzidine (DAB) and examined by light microscopy. RGC densities were determined by counting labelled cells in three areas (62 500 mm^2^) per retinal quadrant at eccentricities of 1/6, 3/6 and 5/6 of the retinal radius. Two independent investigators, who were blinded to the protocol, performed the cell counts.

### Statistics

ANOVA was used to compare retinal thickness, myelin sheath number, ganglion cell number, VEP P2 latency time and ERG amplitude at different times in infected versus control eyes. Student’s *t* -tests were used to compare retinal thickness, myelin sheath number, ganglion cell number, VEP P2 latency time and ERG amplitude of the treated (infected, treated) group to the control (infected untreated) group. Statistics were performed using IBM SPSS statistics 19 (SPSS Inc, USA).

## Results

### *A. cantonensis-*infected mice developed significant retinal and optic nerve inflammation as well as optic nerve demyelination

**Figure 1:**
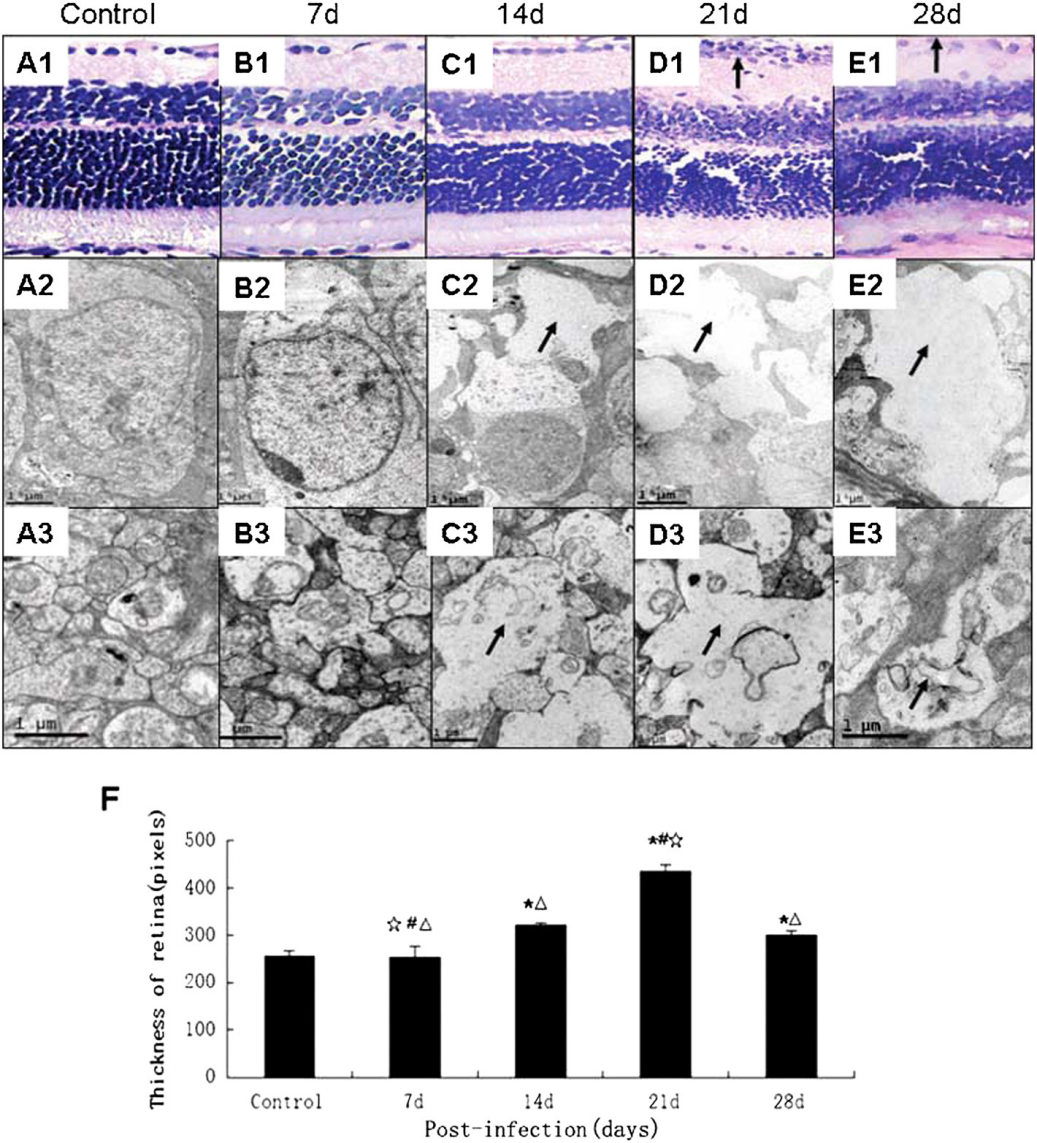
**Retinal histopathology caused by *A. cantonensis*. (A1-E1)**: Retinas from mice infected with *A. cantonensis* for 0 d, 7 d, 14 d, 21 d, and 28 d, respectively. The ganglion cell layer shows inflammatory cell infiltration (black arrow in **D1**) at day 21. The inflammation has decreased by day 28 **(E1)**. Scale bar = 100 mm. **(A2-E2)**: The ganglion cells of mice infected with *A. cantonensis* for 0 d, 7 d, 14 d, 21 d, and 28 d, respectively observed with a scanning electron microscope. On day 14, obvious swelling can be observed in the cytoplasm of ganglion cells (black arrow in **C2**) and becomes more pronounced on day 21 (black arrow in **D2**). By day 28 the swelling has decreased (black arrow in **E2**). Scale bar = 1 μm. **(A3-E3)**: The inner nuclear layer of mice infected with *A. cantonensis* for 0 d, 7 d, 14 d, 21 d, and 28 d respectively. Inner nuclear layer swelling is apparent on day 14 (black arrow in **C3**) and was more serious on day 21 (black arrow in **D3**). Swelling had decreased by day 28 (black arrow in **E3**). Scale bar = 1 μm. **(F)**: Retinal thickness, as seen under a light microscope, in control, 7 d, 14 d, 21 d, and 28 d respectively after infection with *A. cantonensis.* Data are mean ± SEM. *Statistically significant when compared with control (0 d infection) (P < 0.05); # Statistically significant when compared with infection for 14 d (P < 0.05); △ Statistically significant when compared with infection for 21 d; ☆Statistically significant when compared with infection for 28 d. Scale bar = 0.5 μm.

**Figure 2:**
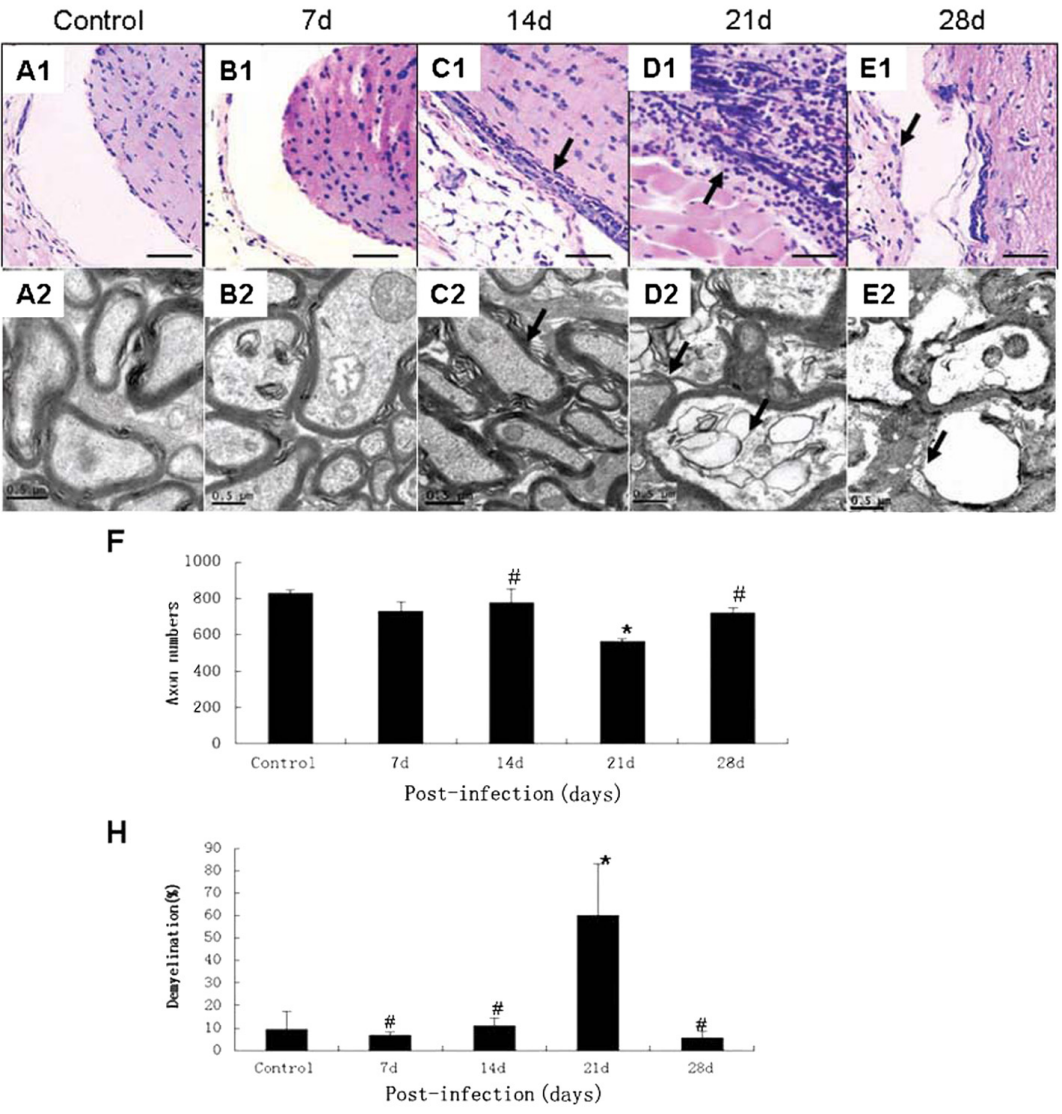
**Histopathological alteration of optic nerve at a variety of time intervals in mice infected with *A. cantonensis*. (A1-E1)**: HE staining of optic nerve infected with *A. cantonensis* for 0 d, 7 d, 14 d, 21 d, and 28 d, respectively. On day 14, obvious inflammatory cell infiltration in the adventitia can be seen and the inflammation became more pronounced (black arrows in **C1** and **D1**) on day 21. Inflammatory cell infiltration had decreased by day 28 and the optic nerve appeared to be atrophied compared with the other groups (gray arrow in **E1**). Scale bar = 40 mm. **(A2-E2)**: Electronic photo of optic nerve infected with *A. cantonensis* for 0 d, 7 d, 14 d, 21 d, and 28 d, respectively. On day 14, demyelination began to appear (black arrows in **C2**); on day 21, obvious demyelination and swelling can be observed (black arrows point in **D2**) and on day 28, an obvious cavity appeared in the myelin sheath (black arrows in **E2**). Scale bar = 0.5 μm. **(F)** Data are mean ± SEM of axon number of 0.58 mm^2^ in control, 7 d, 14 d, 21 d, and 28 d, respectively after infection with *A. cantonensis s.***(H)** Data are mean ± SEM of demyelination rates in control, 7 d, 14 d, 21 d, and 28 d, respectively after infection with *A. cantonensis.* *Statistically significant as compared with control (0 d infection) (P < 0.05); #Statistically significant as compared with infection for 21 d (P < 0.05).

Histopathological examination showed inflammatory cell infiltration in the ganglion cell layer of the retina (Figure 1D1) at 21 days post-infection with *A. cantonensis* though the cellular infiltrate appeared to have subsided at 28 d (Figure 1E1). The results by using an electron microscope showed obvious swelling in the ganglion cells and fibers in the inner nuclear layer at 14 d after infection (Figure 1C2 and C3). At 21 d and 28 d, there was destruction of ganglion cells and inner nuclear layer structures (Figure 1D2 and E2). Retinal thickness continued to increase post-infection and peaked at 21 d. However, at 28 d post-infection retinal thickness highly decreased (Figure 1F). The results of histopathological examination revealed obvious inflammatory cell infiltration around the adventitia of optic nerves at 14 d and 21 d (Figure 2C1 and D1), though this phenotype decreased by 28 d (Figure 2E1). Demyelination began to appear (Figure 2C2) and became apparent at 21 d (Figure 2D2) and 28 d, cavities of different sizes could be observed in the optic nerve (Figure 2E2). The number of axons decreased and the optic nerve was about 60% demyelinated at 21 d (Figure 2F).

### *A. cantonensis-*infected mice had retinal ganglion cell loss

**Figure 3:**
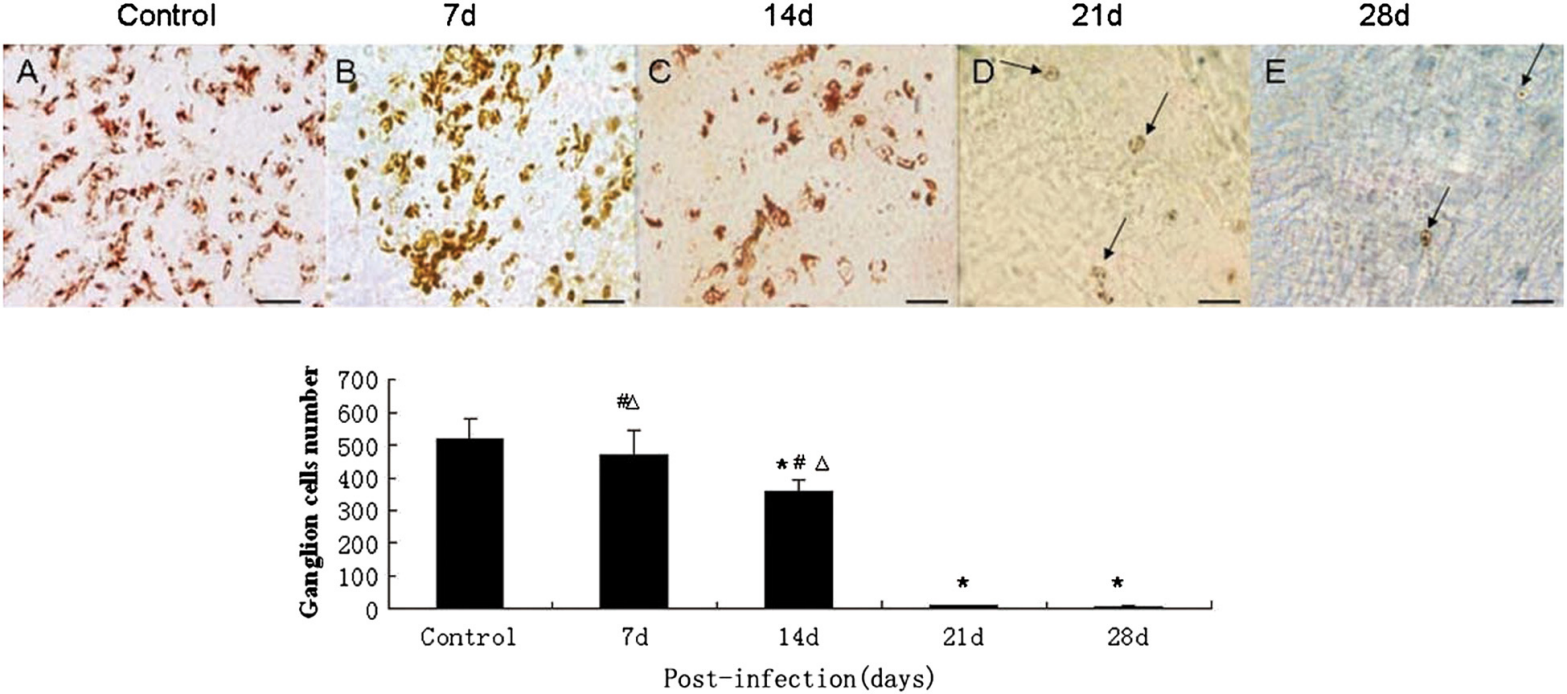
**Number of surviving retinal ganglion cells of the mice infected by *A. cantonensis *at various times. (A-E)** Surviving ganglion cells after infection with *A. cantonensis* for 0 d, 7 d, 14 d, 21 d, and 28 d, respectively. Number of surviving ganglion cells decreased as the length of infection time increased. On days 21 and 28, fewer ganglion cells can be seen (black arrows in **D** and **E**). Scale bar = 40 mm. **(E)** Data are mean ± SEM of retrograde labelled RGCs/mm2 in control, 7 d, 14 d, 21 d, and 28 d, respectively after infection with *A. cantonensis.* *Statistically significant when compared with control (0 d infection) (P < 0.05); # Statistically significant when compared with infection for 21 d (P < 0.05); △Statistically significant when compared with infection for 28 d (P < 0.05).

In histological sections, ganglion cells appeared to be swollen even when the structure had been destroyed. At the same time, the number of axons in the optic nerve decreased and there was evident demyelination. The amount of retinal ganglion cells was counted, after retrograde labeling, to evaluate whether optic nerve function was damaged after *A. cantonensis* infection. The amounts of retinal ganglion cells decreased as extending the infection (Figure 3F) and only a few cells remained alive at day 21 and 28 (Figure 3D and E).

### *A. cantonensis-*infected mice developed optic neuritis in functional assessment of optic nerves and ganglion cells

**Figure 4:**
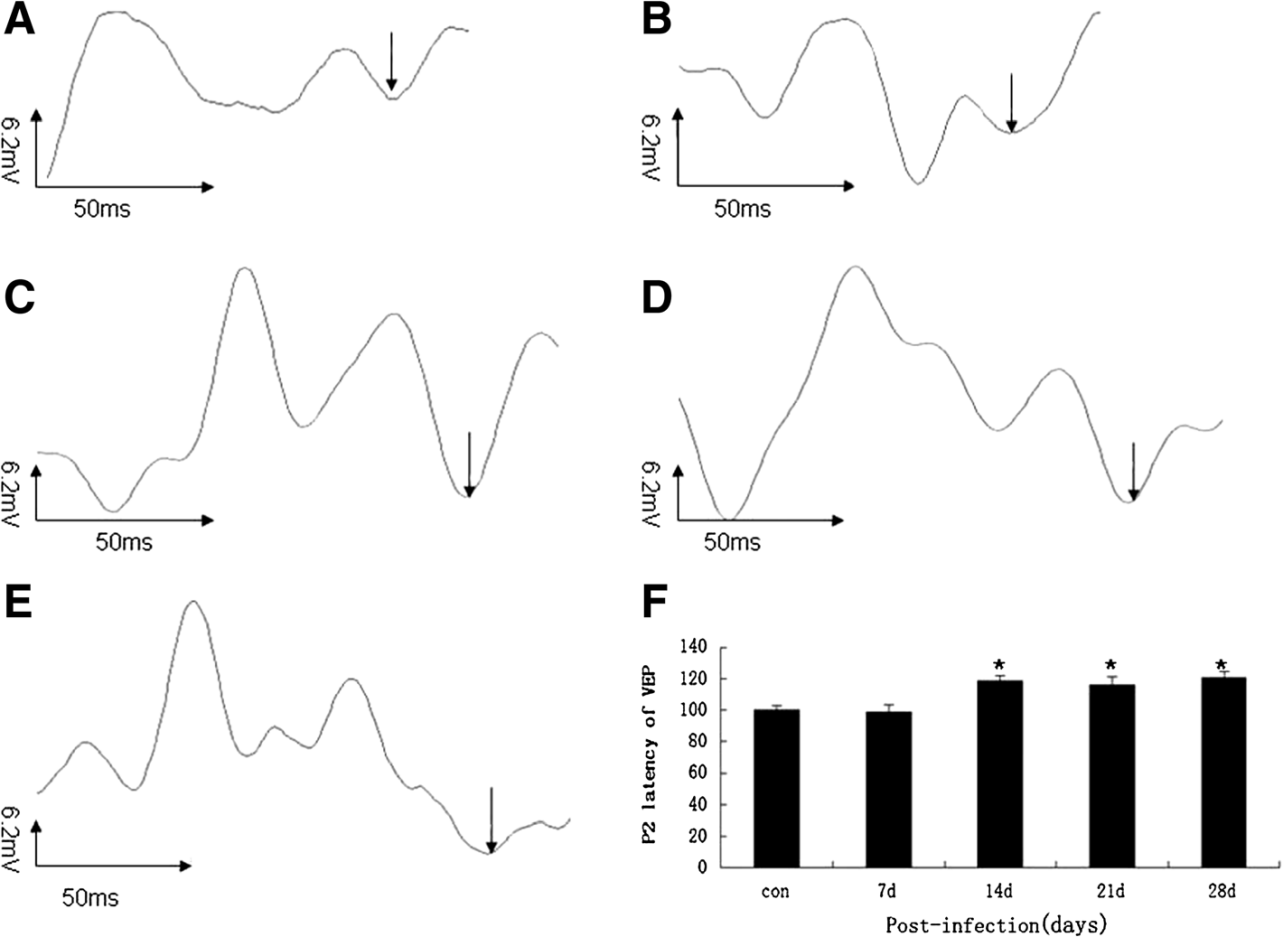
**P2 latency of VEP alteration at various times in mice infected with *A. cantonensis. *(A-E)**: P2 latency of VEP after infection with *A. cantonensis* for control, 7 d, 14 d, 21 d, and 28 d, respectively. **(F)**: Data are mean ± SEM of P2 latency of VEP in control, 7 d, 14 d, 21 d, and 28 d, respectively after infection with *A. cantonensis.* *Statistically significant when compared with control (0 d infection) (P < 0.05).

**Figure 5:**
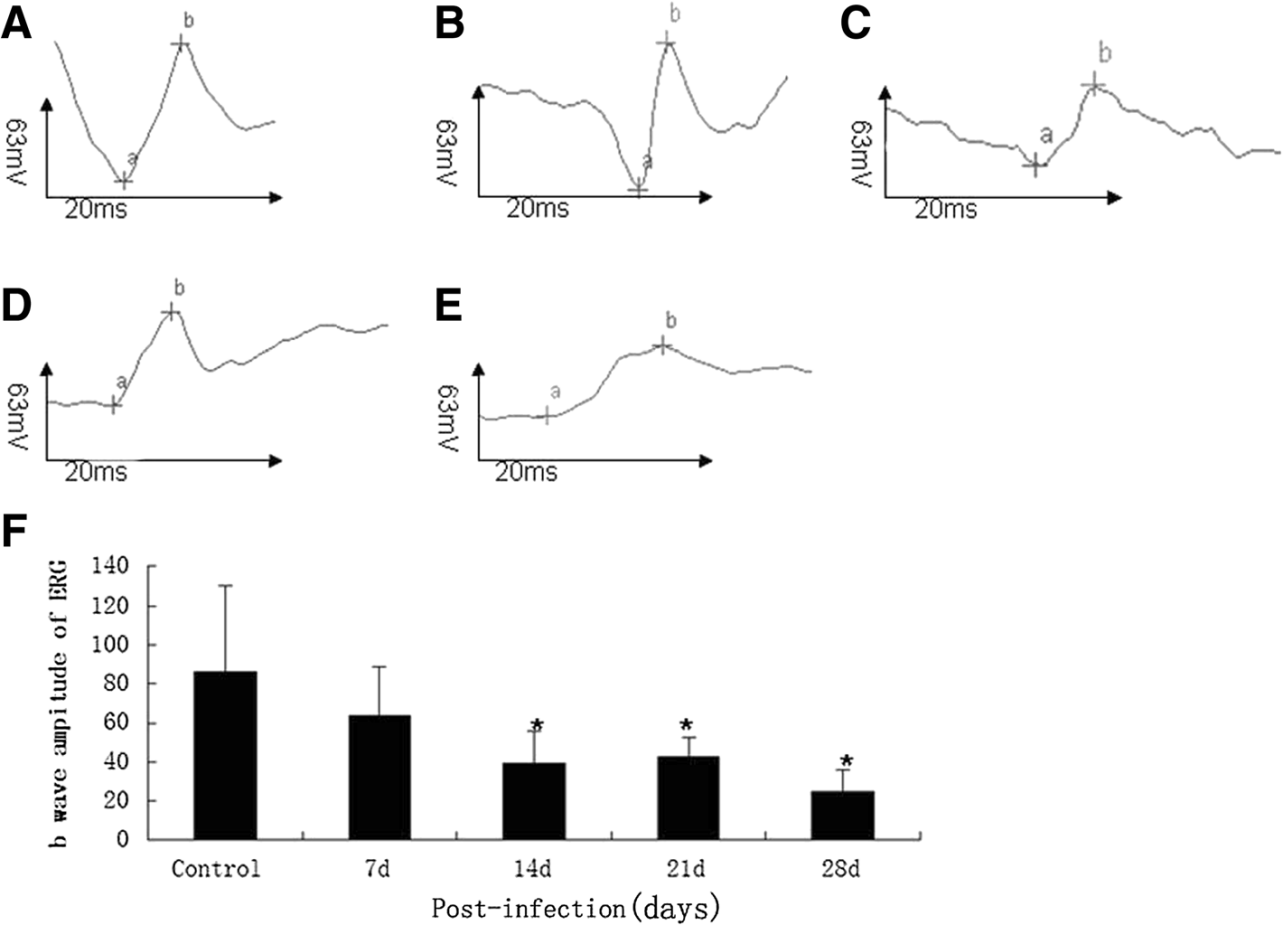
**b wave amplitude of ERG alteration in the mice infected with *A. cantonensis*. (A-E)**: b wave amplitude of ERG after infection with *A. cantonensis* for 0 d, 7 d, 14 d, 21 d, and 28 d, respectively. **(F)**: Data are mean ± SEM of the b wave amplitude of the ERG in control, 7 d, 14 d, 21 d, and 28 d, respectively after infection with *A. cantonensis.* *Statistically significant as compared with control (0 d infection) (P < 0.05).

VEP and ERG measurements were performed in response to flash and pattern stimulation to diagnose optic neuritis *in vivo* and to monitor the function of RGCs. Flash VEP tested the axonal signaling of the optic nerve and ERG measurements in response to flash stimulation determining the function of the retina as a whole [13]. In the present study, the VEP and ERG data showed abnormal change at day 14 post-infection (Figures 4 and 5), compared to normal controls. This result, indicates that infection of *A. cantonensis* indeed affected the axonal signaling of the optic nerve, leading to optic neuritis and the retinal function of *A. cantonensis*-infected mice also was affected.

### TBD therapy reduces inflammation in optic neuritis cases, but had no obvious effect on the functional recovery of vision

**Figure 6:**
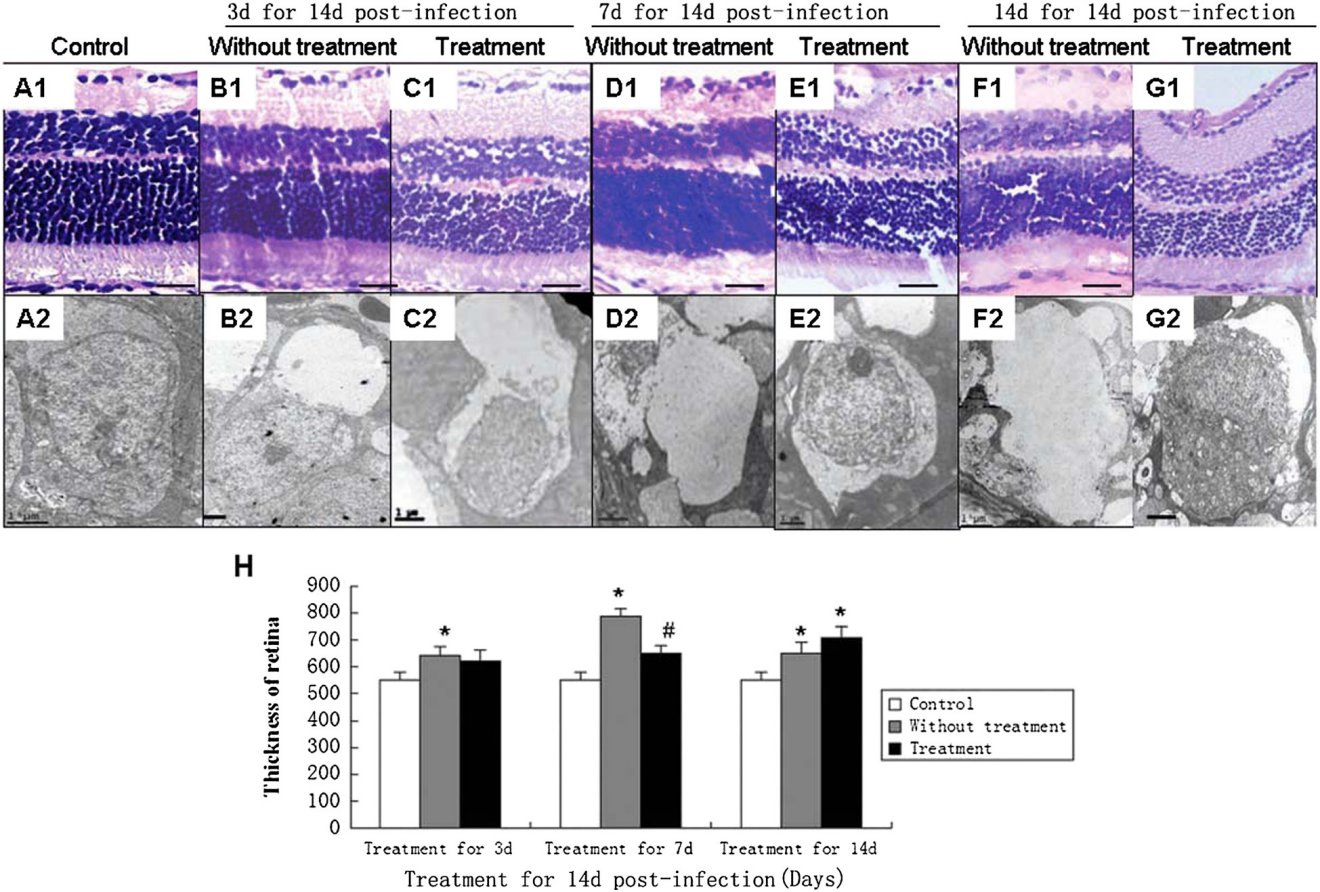
**Histopathologic alteration of the retina in the *A. cantonensis*-infected mice after TBD treatment. (A1-A2)**: Normal retinas and ganglion cells of mice. **(B1-B2)**: Retina and ganglion cells in untreated mice infected with *A. cantonensis* for 14 d; **(C1-C2)**: Retina and ganglion cells in mice treated with TBD for 3 d, after having been infected with *A. cantonensis* for 14 d; **(D1-D2)**: Retina and ganglion cells in untreated mice infected with *A. cantonensis* for 21 d; **(E1-E2)**: Retina and ganglion cells in mice treated with TBD for 7 d having been infected with *A. cantonensis* for 14 d; **(F1-F2)**: Retinas in untreated mice infected with *A. cantonensis s* for 28 d; **(G1-G2)**: Retina and ganglion cells in mice treated with TBD for 7 d after having been infected with *A. cantonensis* for 14d (**A1-G1**: HE staining, bar = 100 μm; **(A2-G2)**: electronic photo, bar = 1 μm). **(H)**: Data are mean ± SEM of retina thickness with or without TBD treatment for 3 d, 7 d, and 14 d. *Statistically significant when compared with control (0 d infection) (P < 0.05); #Statistically significant when compared with untreated groups (P < 0.05).

**Figure 7:**
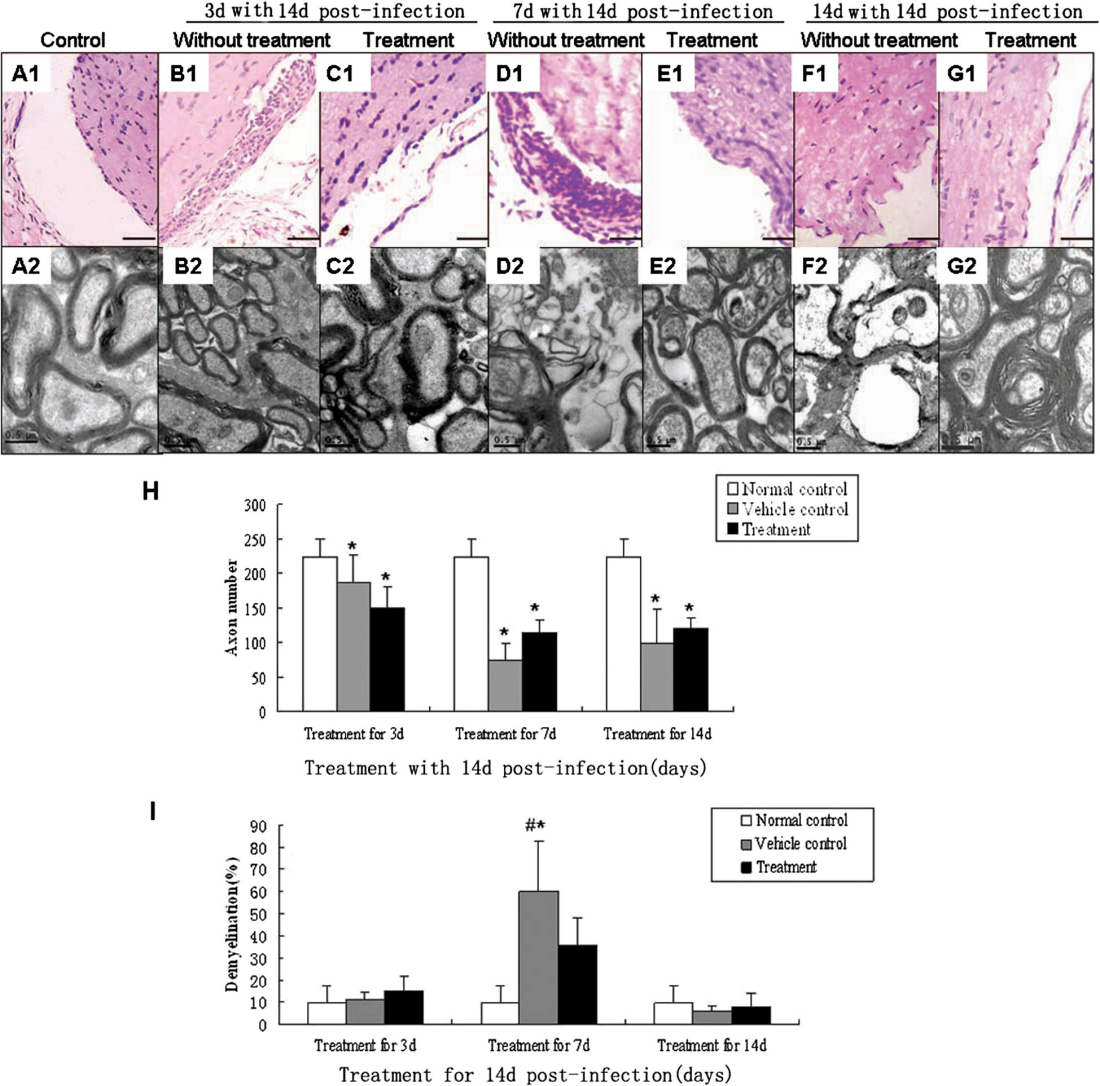
**Histopathological alteration of the optic nerve in the *A. cantonensis*-infected mice after TBD treatment. A1-G1**: HE staining, bar = 100 μm; **A2-F2**: electronic photo, bar = 0.5 μm. **(A1-A2)**: Normal optic nerve of mice **(B1-B2)**: Optic nerve in untreated mice infected with *A. cantonensis* for 14 d; **(C1-C2)**: Optic nerve in mice treated with TBD for 3 d, after 14 d of *A. cantonensis* infection; **(D1-D2)**: Optic nerve in untreated mice infected with *A. cantonensis s* for 21 d; **(E1-E2)**: optic nerve in mice treated with TBD for 7 d after 14 d of *A. cantonensis* infection; **(F1-F2)**: Optic nerve in untreated mice infected with *A. cantonensis* for 28 d; **(G1-G2)**: Optic nerve in mice treated with TBD for 14 d after 14 d *A. cantonensis* infection. **(H)**: Data are mean ± SEM of axon numbers with or without TBD treatment for 3 d, 7 d, and 14 d, respectively. **(I)** Data are mean ± SEM for the demyelination rate with or without TBD treatment for 3 d, 7 d, and 14 d, respectively. *Statistically significant when compared with control (0 d infection) (P < 0.05).

**Figure 8:**
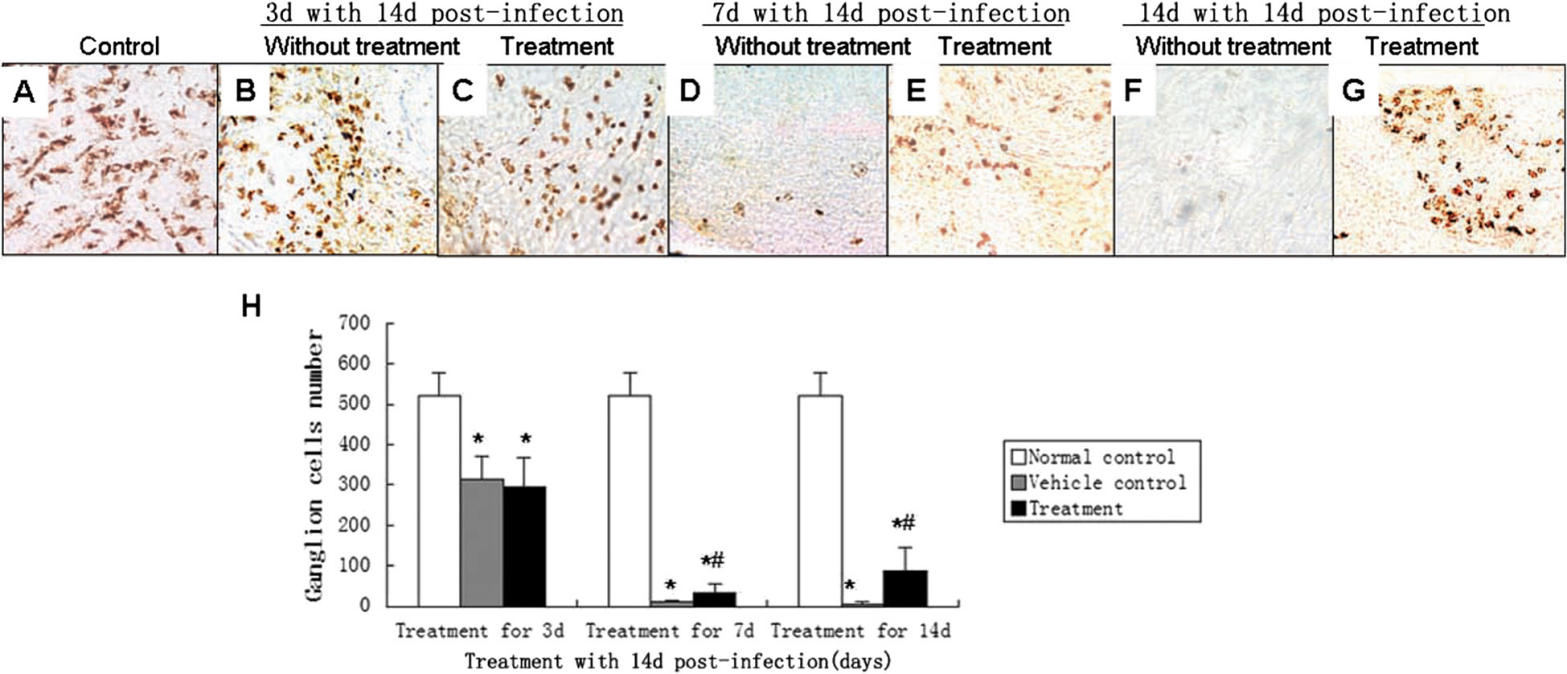
**TBD promotes RGC survival of mice infected with *A. cantonensis*. (A)** RGC cells from normal controls labeled with BDA **(B)** Representative whole-mount area at three-sixths of the retinal radius. On day 17 after infection, RGCs in untreated eyes are labelled with BDA. **(C)** After treatment with TBD for 3 d, there was no obvious difference compared to the untreated group. **(D)** Infection for 21 d without treatment. The RGCs induced much less than control. **(E)** After treatment with TBD for 7 d, the number of RGCs increased compared to the untreated group **(D)**. **(F)** Infection for 28 d without treatment. The RGCs induced much less than control **(G)**. After treatment with TBD for 14 d, the number of RGCs increased compared to the group without treatment **(F). (H)** Data are mean ± SEM for retinal thickness with or without TBD treatment for 3 d, 7 d, and 14 d, respectively. *Statistically significant when compared with control (0 d infection); #Statistically significant when compared to untreated groups (P < 0.05).

**Figure 9:**
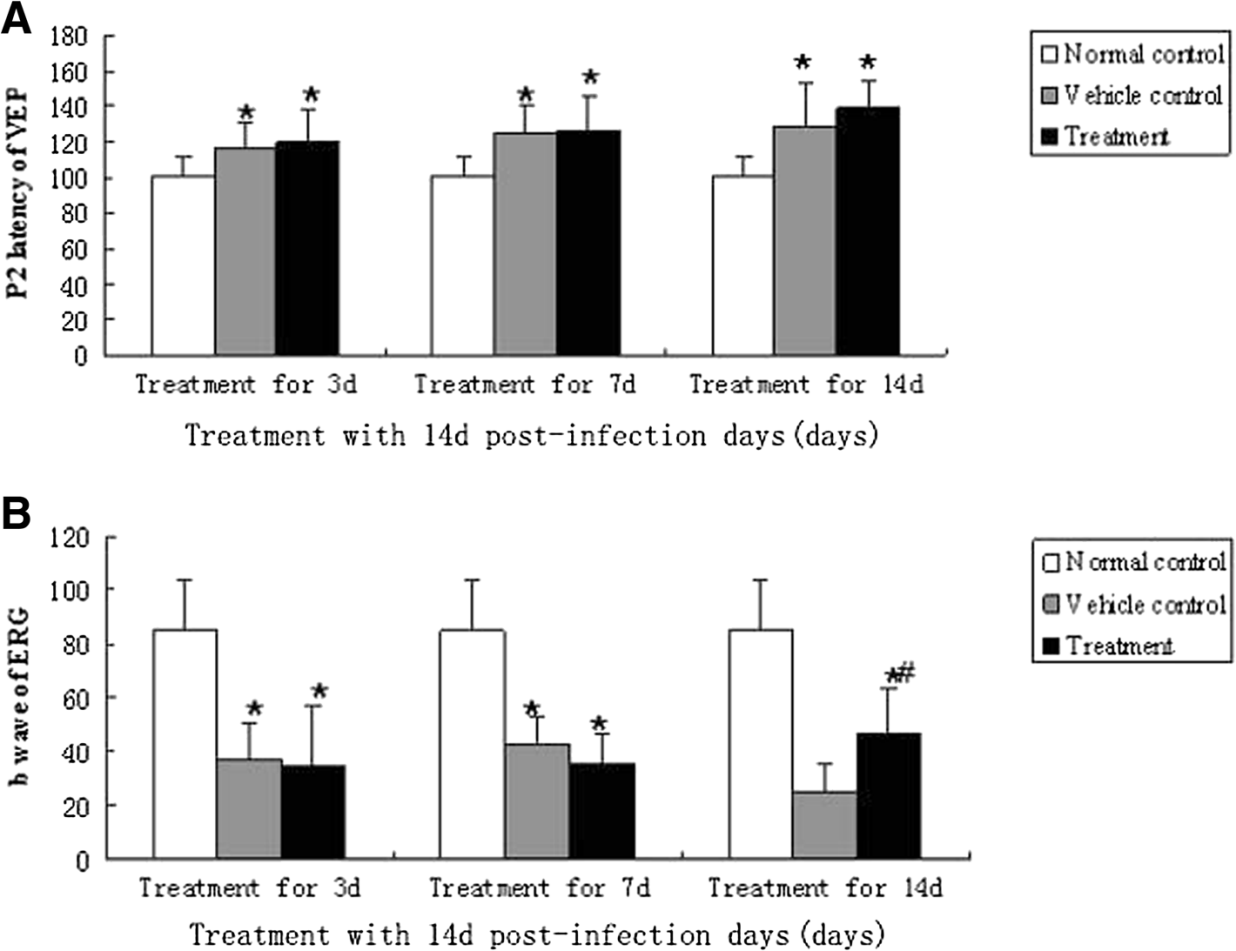
**VEP and ERG alterations of mice infected with *A. cantonensis. *(A)** Data are mean ± SEM for P2 latency alterations of VEP in normal, vehicle control and treated mice at 3 d, 7 d and 14 d after infection. There was no difference in infected, treated and the infected, untreated groups at various times. However, in the infected, treated and infected, untreated groups, there are obvious differences compared to normal controls. **(B)** Data are mean ± SEM for b wave amplitude of ERG in normal, vehicle control and treated mice at 3 d, 7 d and 14 d. after 3 and 7 d of treatment, there was no difference between treated-groups and untreated-groups. Both treated group and untreated-group had an obvious decrease in b wave amplitude compared to normal controls. After 14 d of treatment, amplitude of the b wave in the treated group had increased compared to the untreated group, though b wave amplitude still not recovered to a normal level in the treated group. *Statistically significant when compared with normal control (P < 0.05); #Statistically significant when compared with groups without treatment (P < 0.05).

Histological examination of the retina and optic nerve, retinal ganglion cell counts, as well as VEP and ERG measurements were carried out to estimate the therapeutic effect of TBD treatment. After TBD treatment on 3, 7 and 14 days within the period of 14 days post-infection, mice in the treatment groups were examined and compared with mice in the normal control and vehicle control groups. Retinal inflammation subsided at day 3 and 7 and the retina was almost fully recovered at day 14 of treatment (Figure 6C1-G1).Ganglion cell swelling had also decreased at day 7 and 14 (Figure 6E2-G2) with TBD treatment for 7 days at 14 days post-infection. Retinal swelling was alleviated and the retina was thinner, compared to control groups without treatment. However, there was no difference between the group that was treated for 14 d and the group that did not receive treatment and neither group recovered normal retina thickness (Figure 6H).Optic nerve inflammation was also reduced after the treatment for 3 and 7 days (Figure 7B1-E1). Demyelination of the optic nerve was decreased after treatment for 7 days (Figure 7D2, E2, I) and cavities were no longer observed in nerve fibers of eyes at day 14 in the treatment group compared to the untreated group (Figure 7F2, G2). The optic nerve demyelination rate had also recovered to a normal level (Figure 7H, I).The surviving ganglion cells were counted, and ERG and VEP measurements were carried out to estimate the functional recovery of ganglion cells and optic nerve after TBD treatment. The amounts of surviving ganglion cells had increased after treatment for 7 days and 14 days within 14 days post-infection (Figure 8), compared to the vehicle control group. However, there was still an obvious difference when compared to the amounts of ganglion cells in eyes from the normal control group. On the other hand, VEPs in the treated groups were not any better than untreated-groups at any time (Figure 9A). The ERG had improved after treatment for 14 days within 14 days post-infection (Figure 9B), but had still not recovered to a normal level.

## Discussion

Mice and human beings are both non-permissive hosts of *A. cantonensis* and infection with this nematode are shown to cause similar, serious central nervous system (CNS) symptoms in both. Therefore, mice have been commonly selected as an animal model to study the infection caused by *A. cantonensis*[16,17]. In the present study, BALB/c mice were chosen as the animal model since they are more sensitive to *A. cantonensis* than other types of mice. Although there are clinical reports and experimental observations illustrating that *A. cantonensis* can cause optic neuritis in human beings and mice, there have been no systematic studies examining optic neuritis caused by *A. cantonensis*. Therefore, we carried out the systematic experiments by utilizing the mice in order to observe and treat the optic neuritis.

Clinically, the most common techniques used to diagnose optic neuritis caused by *A. cantonensis* are funduscopic examination and ERG and VEP combined with immunologic diagnosis by ELISA and/or specific antibody detection in serum [18,19], though larval detection in the eyes is still regarded as the most reliable method [16]. Histological examination of the retina and optic nerve are always used as an index for studies on pathogenic alterations due to optic neuritis, such as in EAE model [20].

In the present study, we utilized histological examination, such as H&E staining, employed transmission electronic microscopy and ERG/VEP testing to measure optic neuritis. Many inflammatory cell infiltrates were observed with H&E staining in the retinal ganglion cell layer and around the adventitia of the optic nerve at day 14 of infection. The retinal thickness increased in infected eyes, compared to normal controls, and peaked at day 21, which indicates that swelling may occur in the retina. Similarly, the result of electron microscopy examination showed swelling of the ganglion cells and internal nuclear layer. Interestingly, the retinal swelling subsided at day 28 after infection. The electron microscope examination of ganglion cells showed that the organelle vanished, which may be a reason for the thinner retina observed at day 28. On the other hand, the numbers/mm^2^ of axons in the optic nerve decreased at day 14 and obvious demyelination appeared at day 21. This indicates that the *A. cantonensis* infection seriously affected the optic nerve, resulting in optic neuritis since demyelination is a common cause of optic neuritis. Shed myelin slows optic signal transduction causing an obvious visual disorder [21].

Ganglion cells are the most important neurons in the retina and their axons form the optic nerve. It is well known from the rat model of surgical axotomy of the optic nerve that 80 – 90% of retinal ganglion cells undergo apoptotic cell death following optic nerve transection [22,23]. Inflammatory demyelination induces axonal injury and retinal ganglion cell apoptosis in experimental optic neuritis [24]. The BDA retrograde labelling result indicated that some ganglion cells were lost and that the optic nerves had some demyelination, which provides evidence of the occurrence of optic neuritis in *A. cantonensis* infected mice.

It is known that RGC loss can occur before alterations in VEP potentials, which is detectable. However, when RGC death has reached a certain threshold, ERG potentials become affected and severe functional deficits in VEP responses occur [25]. In the present study, at day 14 the VEP latency of infected animals was obviously delayed and the b wave amplitude of the ERG had apparently decreased. This may be closely correlated with damage to the optic nerve fibers and retinal ganglion cells, which provides the convincing evidence that *A. cantonensis* infection can cause optic neuritis.

The evidence so far points to the fact that the infection of *A. cantonensis* can lead to optic neuritis. However, preventing and/or treating this disease is still a problem. In clinic, physical removal of the parasites via surgery, oral steroid therapy combined with an anthelmintic agent is the most effective therapy for the vision damage caused by parasites. It is because this treatment reduces intraocular inflammation and improves visual acuity to a certain extent [26]. In some cases, there is no obvious improvement in vision even after treatment with a steroid [10]. Therefore, we investigated whether TBD, a new anthelmintic agent, is able to treat the disease. TBD has a rapid anthelmintic action, a lower toxicity, and no mutagenic or teratogenic effects [27]. The inflammation of the retina and optic nerve was quickly reduced, and the number of surviving ganglion cells and the axon numbers/mm^2^ in the optic nerve increased in mice of the TBD-treated groups, compared to untreated groups. It may be that TBD killed *A. cantonensis* and lessened the eosinophil infiltration in the brain and optic nerve caused by this type of parasite, which in turn, decreased the demyelination of the optic nerve and reduced the death of ganglion cells. At day 14, the ERG in the TBD treatment group had recovered better compared with the untreated group, though they had still not reached a normal level. The better ERG recovery may be correlated with the increase in surviving ganglion cells. On the other hand, there was no obvious VEP recovery with TBD treatment. This is likely due to the fact that there were not enough surviving ganglion cells or optic nerve fibers to reach a certain threshold where they can function normally. This result is similar to what is observed clinically in cases of optic neuritis when other medicines are applied [9]. But it also indicates that only TBD treatment is not enough for this disease because it cannot protect the optic nerve during *A. cantonensis* infection treatment and can not reverse the serious damage of ganglion cells and optic nerve effectively. As a result, other therapy methods for demyelination may be drawn, such as Multiple sclerosis (MS).

MS is the most common non-traumatic cause of neurological disability in young adults in developed countries. It is an autoimmune CNS disease that has long been thought to be primarily characterized by inflammation and demyelination [28]. The disease usually begins with attacks of neurological dysfunction, such as loss of vision in one eye (involvement of an optic nerve) and/or weakness (corticospinal tracts of brain or spinal cord). Here, our study demonstrates that the optic neuritis caused by *A. cantonensis* results in pathological alterations and clinical manifestations, such as ganglion cell loss, demyelination and prolonged VEP latency, which are similar to MS [20]. This implies that the optic neuritis caused by *A. cantonensis* or by MS may have some connecting links. It has been reported that helminthes may be a potential immunological therapy for MS. Helminthes invade a host and induce abnormal immunoregulation which could be used to treat MS [29]. *A. cantonensis* is a type of helminth, but it invades the CNS directly causing symptoms similar to MS, thus it may aggravate MS instead of treating it. Although the nosogenesis of both diseases are very different, the methods to relieve symptoms used in MS therapy, such as combining neuroprotective agents with Dexamethasone treatment and interferon treatment, may provide new ways to treat optic neuritis caused by *A. cantonensis*[30-32]*.*

## Conclusions

The above results indicate that *A. cantonensis* infection can cause optic neuritis and that TBD is an effective anthelmintic agent that could be used to treat this disease as it can effectively lessen the symptoms of optic neuritis. However, TBD is not an effective therapy for recovering vision. We suggest that TBD combined with a neuroprotective agent and Dexamethasone may provide better and more effective treatment for optic neuritis caused by *A. cantonensis*. Combining these treatments may help to prevent secondary injuries caused by *A. cantonensis* and may lessen other serious sequelae in the ocular region, such as visual impairment even vision loss. This will ultimately improve the quality of life for patients who suffer from this type of disease. Finally, due to the similarity of *A. cantonensis* and MS symptoms, therapies used for MS may also be helpful for treating optic neuritis caused by *A. cantonensis.*

## Abbreviations

TBD: Tribendimidine; ANOVA: Analysis of variance; A *cantinensis*: *Angiostrongylus cantonensis*; VEP: Visual evoked potential; ERG: Electroretinogram; HRP: Horseradish peroxidase; BDA: Dextran biotin; CNS: Central nerve system; RGC: Retina ganglion cells; MS: Multiple sclerosis.

## Competing interests

The authors declare that they have no competing interests.

## Authors’ contributions

YF, XZ and LO carried out the experiments and performed the statistical analyses. YF and ZW drafted the manuscript. WL, WW helped to do the ophthalmologic examination section of the study. WC and FF provided help in animal experiment. ZW conceived the study and coordinated the project. XS helped to draft the manuscript. All authors read and approved the final manuscript.

## References

[B1] Diao Z Wang J Qi H Li X Zheng X Yin C Human ocular angiostrongyliasis: a literature review Trop Doct 2011 41 76 78 21296846 10.1258/td.2010.100294

[B2] Feng Y Nawa Y Sawanyavisuth K Lv Z Wu ZD Comprehensive review of ocular angiostrongyliasis with special reference to optic neuritis Korean J Parasitol 2013 51 613 619 24516263 10.3347/kjp.2013.51.6.613 PMC3916447

[B3] Ketsuwan P Pradatsu Padilla-Docal B Iglesias-González I Bu-Coifiu-Fanego R Socarrás-Hernández CA Dorta-Contreras AJ Intrathecal activation as a typical immune response within the central nervous system in angiostrongyliasis Am J Trop Med Hyg 2013 88 230 235 23390222 10.4269/ajtmh.12-0151 PMC3583310

[B4] Wang QP Wu ZD Wei J Owen RL Lun ZR Human Angiostrongylus cantonensis: an update Eur J Clin Microbiol Infect Dis 2012 31 389 395 21725905 10.1007/s10096-011-1328-5

[B5] Monte TC Simões RO Oliveira AP Novaes CF Thiengo SC Silva AJ Estrela PC Maldonado A Jr Phylogenetic relationship of the Brazilian isolates of the rat lungworm Angiostrongylus cantonensis (Nematoda: Metastrongylidae) employing mitochondrial COI gene sequence data Parasit Vectors 2012 6 248 256 23130987 10.1186/1756-3305-5-248 PMC3514143

[B6] Diaz JH Helminthic eosinophilic meningitis: emerging zoonotic diseases in the South J La State Med Soc 2008 160 333 342 19283982

[B7] Prommindaroj K Leelawongs N Pradatsundarasar A Human angiostrongyliasis of the eye in Bangkok Am J Trop Med Hyg 1962 1 759 761 13986349 10.4269/ajtmh.1962.11.759

[B8] Liu IH Chung YM Chen SJ Cho WL Necrotizing retinitis induced by Angiostrongylus cantonensis Am J Ophthalmol 2006 141 577 579 16490516 10.1016/j.ajo.2005.09.033

[B9] Shindler KS Ventura E Dutt M Rostami A Ocular angiostrongyliasis: clinical study of three cases Eye (Lond) 2008 22 1446 1448 18535614 10.1038/eye.2008.135

[B10] Wang J Zheng XY Yin CH Qi HY Li XL Diao ZL Wang F Ji AP Feng ML Guo ZZ Clinical Observation on 25 Cases of Severe *Angiostrongyliasis Cantonensis* Chin J Parasitol Parasit Dis 2007 25 333 336 18038807

[B11] Chen JH Wang H Chen JX Bergquist R Tanner M Utzinger J Zhou XN Frontiers of parasitology research in the People's Republic of China: infection, diagnosis, protection and surveillance Parasit Vectors 2012 4 221 230 10.1186/1756-3305-5-221 PMC3497869 23036110

[B12] Wang J Wei J Zeng X Liang JY Wu F Li ZY Zheng HQ He HJ Wu ZD Efficacy of tribendimidine against Angiostrongylus cantonensis infection in the mice Parasitol Res 2013 112 1039 1046 23377146 10.1007/s00436-012-3228-8

[B13] Zeng X Wang J Wei J Wu F Fung F Wu X Sun X Zheng H Lv Z Wu Z Angiostrongylus cantonensis: tegumental and hypodermic alterations of the fourth-stage larvae following administration of tribendimidine in vivo and in vitro Parasitol Res 2013 112 3035 3040 23728774 10.1007/s00436-013-3479-z

[B14] Meyer R Weissert R Diem R Storch MK de Graaf KL Kramer B Acute neuronal apoptosis in a rat model of multiple sclerosis J Neurosci 2001 21 6214 6220 11487644 10.1523/JNEUROSCI.21-16-06214.2001 PMC6763179

[B15] Guo PJ Zhan XM Gan M Pan ZH Yu YJ Zhang MC Qu ZY Li ZY He A Pathological change in the brain of mice infected with Angiostrongylus cantonensis Zhongguo Ji Sheng Chong Xue Yu Ji Sheng Chong Bing Za Zhi 2008 26 353 355 19157298

[B16] Peng H Sun R Zhang Q Zhao J Wei J Zeng X Zheng H Wu Z Interleukin 33 mediates type 2 immunity and inflammation in the central nervous system of mice infected with Angiostrongylus cantonensis J Infect Dis 2013 207 860 869 23148283 10.1093/infdis/jis682

[B17] Sawanyawisuth K Treatment of angiostrongyliasis Trans R Soc Trop Med Hyg 2008 102 990 996 18501934 10.1016/j.trstmh.2008.04.021

[B18] Pojda-Wilczek D Retrospective analysis of pattern VEP results in different ocular and systemic diseases Klin Oczna 2010 112 205 209 21117363

[B19] Quinn TA Dutt M Shindler KS Optic neuritis and retinal ganglion cell loss in a chronic murine model of multiple sclerosis Front Neurol 2011 2 50 21852980 10.3389/fneur.2011.00050 PMC3151613

[B20] Zhao YY Wei SH Recent progress on genetics of optic neuritis Zhonghua Yan Ke Za Zhi 2011 47 1143 1146 22336125

[B21] Isenmann S Wahl C Krajewski S Reed JC Ba¨hr M Up-regulation of Bax protein in degenerating retinal ganglion cells precedes apoptotic cell death after optic nerve lesion in the rat Eur J Neurosci 1997 9 1763 1772 9283831 10.1111/j.1460-9568.1997.tb01534.x

[B22] Diem R Meyer R Weishaupt JH Ba¨hr M Reduction of potassium currents and phosphatidylinositol 3-kinase-dependent Akt phosphorylation by tumor necrosis factor-a rescues axotomized retinal ganglion cells from retrograde cell death in vivo J Neurosci 2001 21 2058 2066 11245689 10.1523/JNEUROSCI.21-06-02058.2001 PMC6762605

[B23] Shindler KS Ventura E Dutt M Rostami A Inflammatory demyelination induces axonal injury and retinal ganglion cell apoptosis in experimental optic neuritis Exp Eye Res 2008 87 208 213 18653182 10.1016/j.exer.2008.05.017 PMC2564281

[B24] Hobom M Storch MK Weissert R Maier K Radhakrishnan A Kramer B Mechanisms and time course of neuronal degeneration in experimental autoimmune encephalomyelitis Brain Pathol 2004 14 148 157 15193027 10.1111/j.1750-3639.2004.tb00047.x PMC8095969

[B25] Hood DC Greenstein VC Multifocal VEP and ganglion cell damage: applications and limitations for the study of glaucoma Prog Retin Eye Res 2003 22 201 251 12604058 10.1016/s1350-9462(02)00061-7

[B26] Beck RW Cleary PA Anderson MM Jr Keltner JL Shults WT Kaufman DI Buckley EG Corbett JJ Kupersmith MJ Miller NR Savino PJ Guy JR Trobe JD McCrary JA Smith CH Chrousos GA Thompson HS Katz BJ Brodsky MC Goodwin JA Atwell CW A randomized controlled trial of corticosteroids in the treatment o f acute optic neuritis. The Optic Neuritis Study Group N Eng l JM Ed 1992 326 581 588 10.1056/NEJM199202273260901 1734247

[B27] Xiao SH Wu HM Wang C Tribendimidine–a new broad-spectrum drug against intestinal helminths Zhongguo Ji Sheng Chong Xue Yu Ji Sheng Chong Bing Za Zhi 2004 22 312 315 15830892

[B28] Noseworthy JH Lucchinetti C Rodriguez M Weinshenker BG Multiple sclerosis N Engl J Med 2000 343 938 952 11006371 10.1056/NEJM200009283431307

[B29] Fleming JO Helminth therapy and multiple sclerosis Int J Parasitol 2013 43 259 274 23298637 10.1016/j.ijpara.2012.10.025

[B30] Blecharz KG Haghikia A Stasiolek M Kruse N Drenckhahn D Gold R Roewer N Chan A Förster CY Glucocorticoid effects on endothelial barrier function in the murine brain endothelial cell line cEND incubated with sera from patients with multiple sclerosis Mult Scler 2010 16 293 302 20203147 10.1177/1352458509358189

[B31] Diem R Sättler MB Merkler D Demmer I Maier K Stadelmann C Ehrenreich H Bähr M Combined therapy with methylprednisolone and erythropoietin in a model of multiple sclerosis Brain 2005 128 375 385 15601662 10.1093/brain/awh365

[B32] Kjølhede T Vissing K Dalgas U Multiple sclerosis and progressive resistance training: a systematic review Mult Scler 2012 18 1215 1228 22760230 10.1177/1352458512437418

